# Investigating telomere length in progeroid syndromes: implications for aging disorders

**DOI:** 10.18632/aging.206255

**Published:** 2025-05-28

**Authors:** Luma Srour, Abeer Qannan, Junko Oshima, Andre Megarbane, Yosra Bejaoui, Nady El Hajj

**Affiliations:** 1 College of Health and Life Sciences, Hamad Bin Khalifa University, Qatar Foundation, Doha, Qatar; 2 Department of Laboratory Medicine and Pathology, University of Washington, Seattle, WA 98195, USA; 3 Department of Clinical Cell Biology and Medicine, Graduate School of Medicine, Chiba University, Chiba, Japan; 4 Department of Human Genetics, Gilbert and Rose-Marie Chagoury School of Medicine, Lebanese American University, Byblos, Lebanon; 5 Institut Jérôme Lejeune, Paris, France; 6 College of Science and Engineering, Hamad Bin Khalifa University, Qatar Foundation, Doha, Qatar

**Keywords:** telomere length, telomere attrition, progeroid syndromes, DNA methylation

## Abstract

Progeroid syndromes are rare genetic disorders that impact patients' health and lifespans and are characterized by symptoms that mimic the normal aging process. Telomere length is one of the aging hallmarks, a phenomenon linked to cellular aging. Telomere attrition was observed in different progeroid syndromes, such as Nijmegen breakage syndrome patients and Werner syndrome, indicating its contribution to the progeroid phenotype. However, whether it is a common feature in all progeroid syndromes is still unclear. Therefore, in this study, we aimed to estimate telomere length using the DNA methylation-based estimator of human telomere length in publicly available DNA methylation data from patients with Werner Syndrome, Hutchinson-Gilford Progeria Syndrome, Berardinelli-Seip Congenital Lipodystrophy type 2, and Dyskeratosis congenita, along with additional data provided by our laboratory from patients with Cerebroretinal Microangiopathy with Calcifications and Cysts and Wiedemann-Rautenstrauch Syndrome. Our findings revealed that certain progeroid syndromes, including classical Werner Syndrome, Berardinelli-Seip Congenital Lipodystrophy type 2, and Dyskeratosis congenita, have significant telomere attrition conversely to Hutchinson-Gilford Progeria Syndrome, Cerebroretinal Microangiopathy with Calcifications and Cysts, Wiedemann-Rautenstrauch Syndrome, and atypical Werner Syndrome. In conclusion, this study addresses a critical gap by providing new insights into the role of telomere attrition across different progeroid conditions. Further research is needed to elucidate the effect of telomere attrition on progeroid syndromes and its implications.

## INTRODUCTION

Aging is an irreversible, time-dependent deterioration of physiological function due to the gradual accumulation of cellular and molecular alterations that ultimately lead to death [1]. Aging has specific molecular hallmarks such as genomic instability, telomere attrition or dysfunction, and epigenetic alterations, among many other hallmarks [2]. These molecular mechanisms extend beyond the normal aging processes as they are also involved in certain premature aging disorders [3]. The discovery of accelerated aging disorders goes way back to 1874 when Dr. Moritz Kaposi reported unusual signs of premature aging associated with a specific disease called Xeroderma Pigmentosum. Then, a few years later, in 1904, the term progeria, a Greek word that means prematurely old, was introduced by Dr. Hastings Gilford when he first described a case that is currently referred to as Hutchinson-Gilford Progeria syndrome (HGPS) [4, 5]. Finally, in 1978, George Martin proposed the term progeroid, which means ‘resembling progeria’, and suggested using this term to describe diseases that exhibit similar symptoms to progeria but are not exactly alike [6]. Progeroid syndromes are defined as a group of rare heterogeneous disorders with various combinations of aging hallmarks [3, 4]. Thus, most of the progeroid syndromes show features of accelerated aging that mimic normal aging but at a more rapid pace. Progeroid syndromes are classified into segmental and unimodal progeroid syndromes depending on the number of organ systems and tissues that show senescent features. Unimodal refers to organ, and tissue-specific accelerated aging, while segmental progeroid syndromes have a broader impact range that reaches several organ and tissue systems [7]. Recently, 32 premature aging syndromes have been identified; however, the exact number of progeroid syndromes is not well-established due to the phenotypic and genetic heterogeneity of these syndromes [4].

The underlying mechanisms of progeroid syndromes are well-characterized. Progeroid syndromes are mostly caused by a single-gene mutation that disrupts a specific biological pathway [5]. According to the impaired pathway, progeroid syndromes can be classified into two main groups. The first group includes progeroid syndromes with defective nuclear envelope elements, such as Hutchinson-Gilford progeria syndrome (HGPS), mandibuloacral dysplasia, and Néstor-Guillermo progeria syndrome. The second group of progeroid syndromes is caused by defective DNA repair mechanisms that lead to genomic instability, a hallmark of aging [2, 8, 9], such as Werner syndrome (WS), Bloom syndrome (BS), Rothmund-Thomson syndrome (RTS), Cockayne syndrome (CS), Xeroderma Pigmentosum (XP), cerebroretinal microangiopathy with calcifications and cysts (CRMCC), and Nijmegen breakage syndrome (NBS) [9]. Dyskeratosis congenita (DKC) and Hoyeraal-Hreidarsson syndrome (HHS) are examples of the second group with disease-causing mutations in genes associated with telomere maintenance, leading to telomere defects. It is important to note that the underlying mechanisms of progeroid syndromes are not always the same as in physiological aging. Nevertheless, understanding the pathogenesis of progeroid syndromes holds significant value as it might lead to discoveries related to physiological aging, such as revealing new genes or pathways [10].

Human telomeres are dynamic regions with thousands of repeated hexameric nucleotide sequences complexed with nucleoproteins near the end of the linear chromosomes. Their function is to cap and protect the DNA during replication [11, 12]. Telomere attrition is a normal aging phenomenon that occurs gradually during DNA replication due to the inability of DNA polymerases to replicate the DNA sequence in the telomere region. It is well known that telomerase, a specific RNA-dependent DNA polymerase, is responsible for replicating this region. However, most human somatic cells have insufficient telomerase expression, which leads to gradual and cumulative telomere shortening during aging [2].

Previous studies have shown that certain progeroid syndromes, such as NBS [13] and HGPS [14], exhibit telomere attrition. Studies have reported significant shortening in these disorders. It was also indicated that most progeroid syndromes with defective DNA repair mechanisms may have shorter telomeres [13]. However, telomere length measurements have not yet been conducted for most progeroid syndromes. Therefore, in this study, we aimed to estimate telomere attrition in several progeroid syndromes to substantiate their claim and uncover whether it is a common hallmark for all progeroid syndromes.

## RESULTS

### Telomere attrition in progeroid syndrome patients

**Figure 1:**
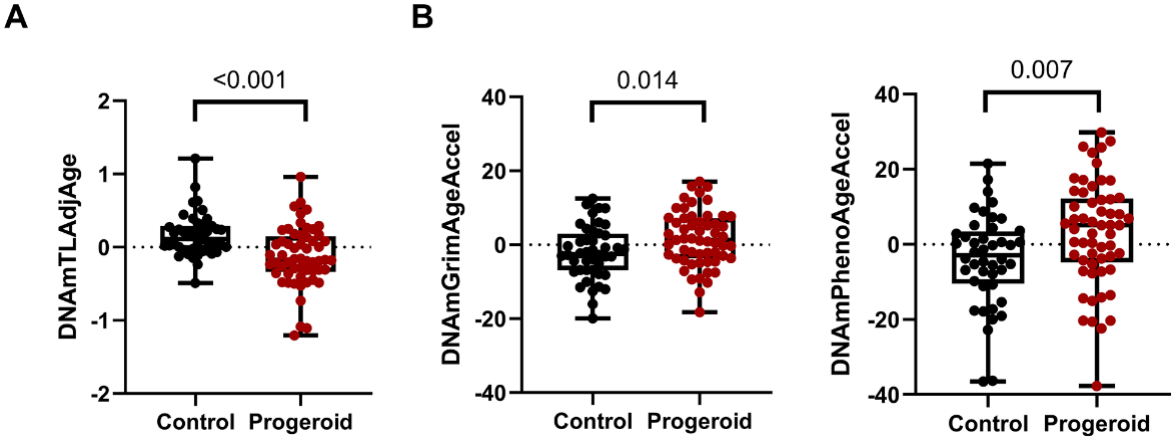
**Comparison between controls and progeroid syndrome patients.** (**A**) Boxplots show significant telomere attrition in patients with progeroid syndromes (n = 57) compared to controls (n = 44), as indicated by the differences in DNAmTLAdjAge. Statistical significance was assessed using a two-tailed Wilcoxon rank-sum test (also known as Mann-Whitney U test) and represented by p-values. (**B**) Epigenetic age acceleration between controls (n = 44) and progeroid syndrome patients (n = 57): Boxplots with individual data points illustrate epigenetic age acceleration, measured using the GrimAge and PhenoAge clocks. Statistical significance was assessed using a two-tailed Student's t-test. All boxplots show the median represented as a line inside the box that represents the interquartile range (IQR), while whiskers extending from the box show the minimum and maximum.

To investigate whether progeroid syndromes have telomere attrition, we calculated telomere length using the DNAmAge web-based calculator. This epigenetic calculator can estimate DNAmTL and several epigenetic ages, such as HorvathAge, HannumAge, GrimAge, PhenoAge, and SkinBloodAge, using DNA methylation data from blood. We calculated the residual of regressing DNA methylation telomere length on actual age to evaluate telomere attrition. At the same time, we calculated the residual of regressing the various epigenetic ages on actual age to assess age acceleration. As a result, significant telomere attrition was observed between the combined progeroid syndromes and their age and gender match controls (*p-value* < 0.001, Figure 1A). In addition, we found a significant age acceleration using GrimAge and PhenoAge between the controls and progeroid groups, as shown in Figure 1B.

**Figure 2:**
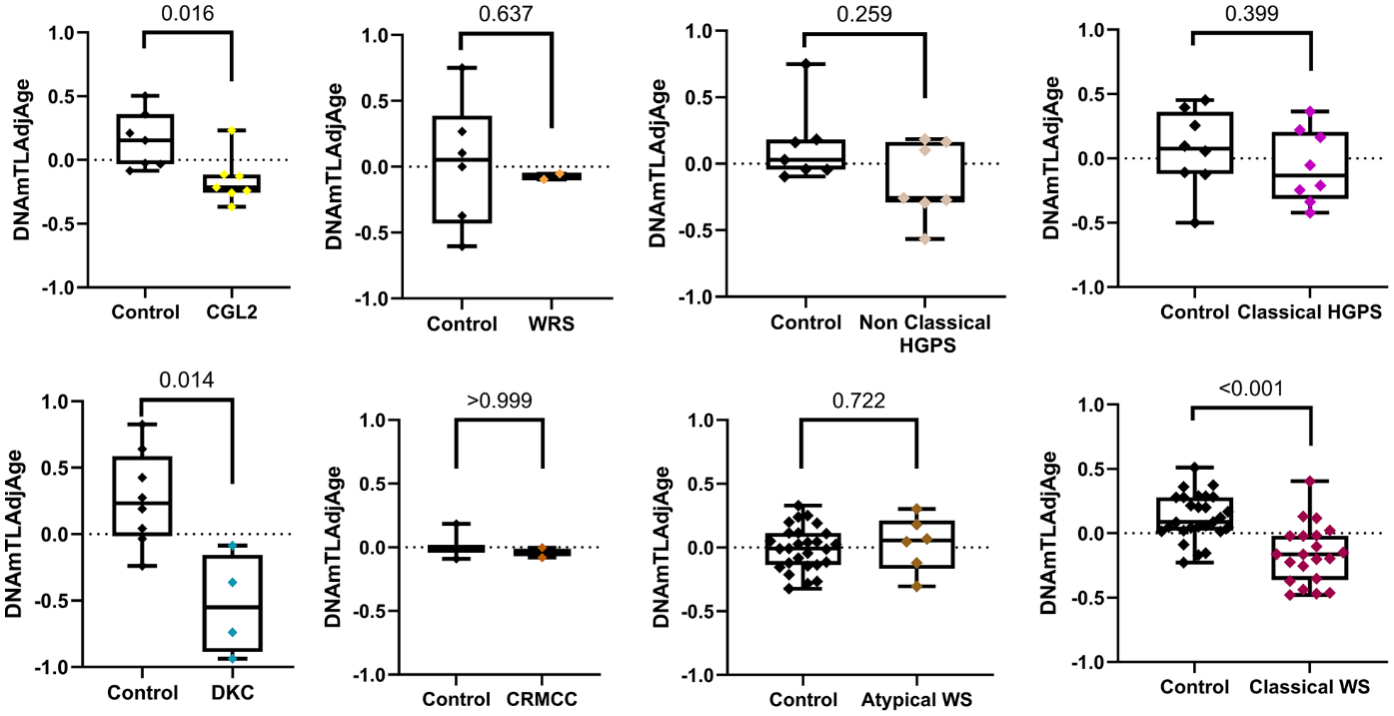
**Boxplots showing DNAmTLAdjAge across various progeroid syndromes compared to controls.** Sample sizes for each group: CGL2 (n = 7), WRS (n = 2), Non-Classical HGPS (n = 7), Classical HGPS (n = 8), DKC (n = 4), CRMCC (n = 2), Atypical WS (n = 6), and Classical WS (n = 21) and controls (CGL2 (n = 7), WRS (n = 6), Non-Classical HGPS (n = 7), Classical HGPS (n = 8), DKC (n = 8), CRMCC (n = 3), Atypical WS (n = 27), and Classical WS (n = 27)). Statistical significance was assessed using a two-tailed Wilcoxon rank-sum test for CRMCC and Non-Classical HGPS, whereas a Student's t-test was used for the remaining comparisons.

Furthermore, we compared patients and age-match controls within each syndrome to investigate which progeroid syndromes have telomere attrition. Significant telomere attrition was observed only in Berardinelli-Seip Congenital Lipodystrophy type 2 (CGL2) (*p* = 0.016), classical WS (*p* < 0.001), and DKC (*p* = 0.014). In contrast, we did not find sufficient evidence to confirm the telomere attrition in HGPS, CRMCC, Wiedemann-Rautenstrauch Syndrome (WRS), and atypical WS, which can be attributed to the low sample size of CRMCC and WRS, as displayed in Figure 2 and Supplementary Figure 1.

### Quantitative PCR reveals significant telomere length reduction in WS and a trend of reduction in CGL2 patients

**Figure 3:**
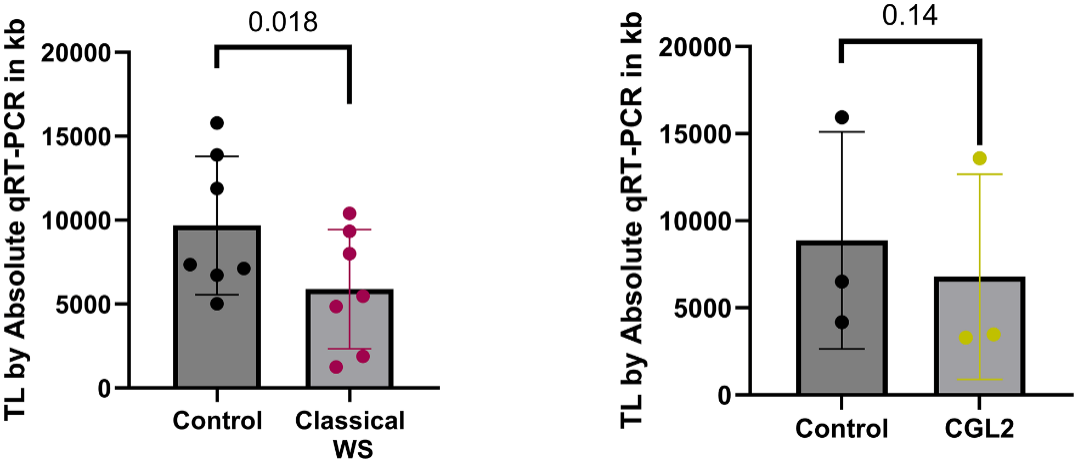
**Comparison of absolute telomere length for WS (n = 7) and CGL2 (n = 3) groups, each analyzed separately against their respective controls (n = 7 and n = 3 for WS and CGL2, respectively).** Telomere length was measured using the Quantitative PCR (qPCR) method. Data are represented as mean +/- SD. Statistical significance was assessed using a two-tailed Wilcoxon rank-sum test and Student's t-test for WS and CGL2, respectively.

To validate changes in telomere length, we used the Absolute Human Telomere Length Quantification qPCR Assay Kit (ScienCell Research Laboratories, CA, USA). We confirmed significant telomere length shortening in patients with Classical WS (N=7) carrying homozygous mutations in the *WRN* gene, compared to age and gender-matched controls (*p =* 0.018, N=7) (Figure 3). However, in CGL2 patients, the tendency of telomere attrition was weaker (*p =* 0.14, Figure 3) due to the low number of samples (N=3) available for validation, as shown in Supplementary Table 2.

### Protective variants have no significant impact on telomere length

To uncover the effect of protective variants on telomere length, we estimated telomere length for individuals carrying protective variants in *APOE, PCSK9*, and *APOC3*. The *APOE* gene has three different alleles associated with Alzheimer's disease (AD). The APOE ε3 allele is known to have a neutral effect on the onset of AD. However, APOE ε4 increases the risk of AD, while APOE ε2 decreases disease risk and delays AD onset [15]. Thus, in this study, we considered individuals carrying the APOE ε3 allele as controls or neutral.

**Figure 4:**
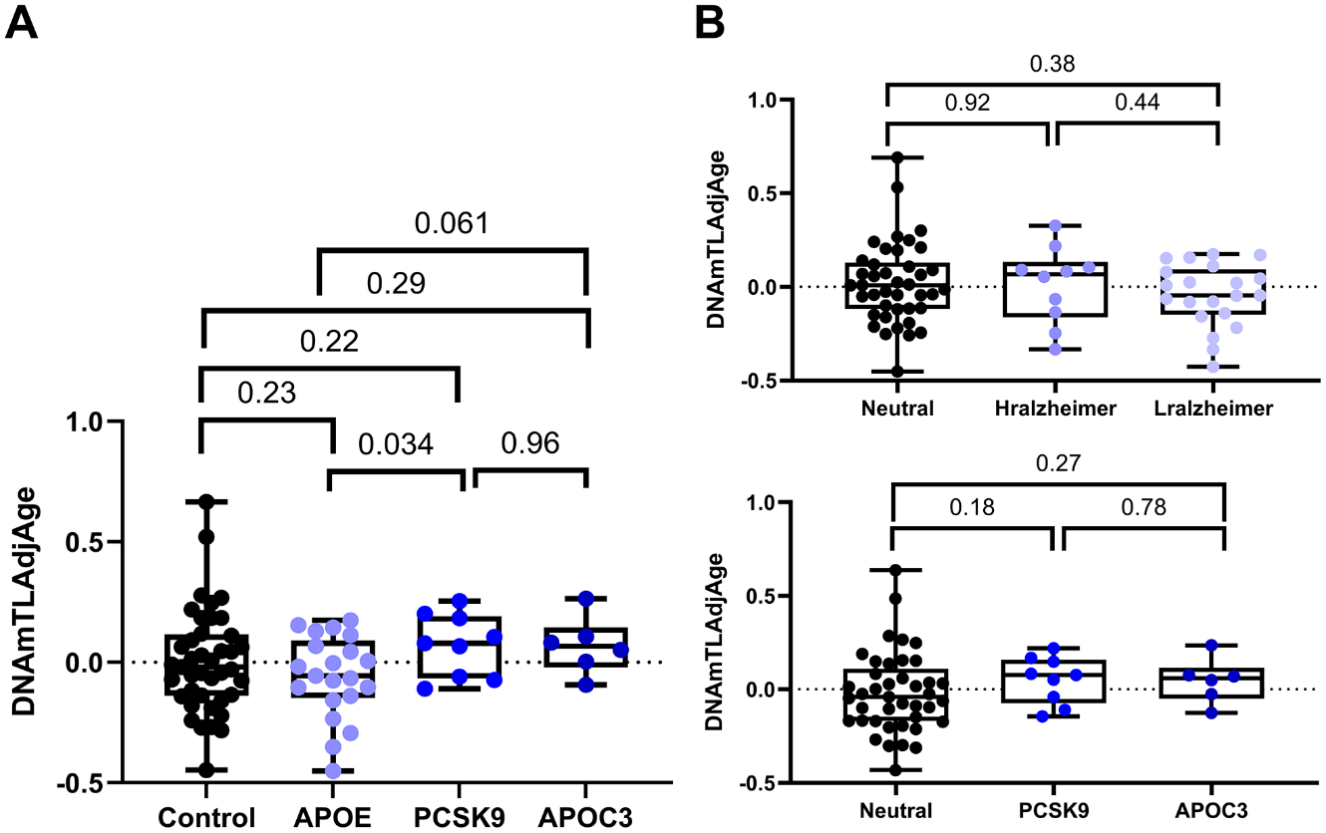
**Comparison of DNAmTLAdjAge across various protective groups.** (**A**) Boxplots illustrate DNAmTLAdjAge across individuals carrying various protective variants. The control group (n = 41) represents individuals with the APOE ε3 allele, while the APOE group (n = 21) includes individuals with the APOE ε2 and APOE ε4 alleles. Sample sizes for the PCSK9 group and APOC3 group were n=9 and n = 6. Statistical significance was assessed using an ANOVA test. (**B**) The upper plot compares DNAmTLAdjAge between the different APOE allele groups (Neutral n = 41, Hralzheimer n = 10, Lralzheimer n = 21). The lower plot compares DNAmTLAdjAge between individuals carrying atherosclerosis protective variants (*PCSK9* n = 9 and *APOC3* n = 6) and 41 controls (with the APOE ε3 allele). Statistical significance was assessed using an ANOVA test.

**Figure 5:**
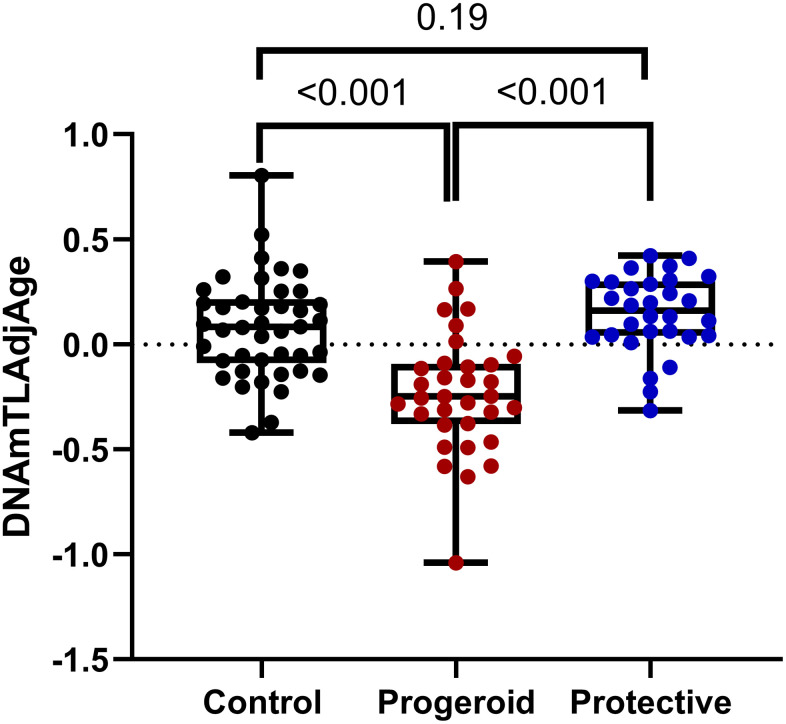
**Comparison of DNAmTLAdjAge across various groups: boxplots illustrate the comparison of DNAmTLAdjAge across different groups, including controls older than 10 years (n = 42), progeroid patients older than 10 years (n = 33), and the protective group (n = 30).** The protective group comprises individuals carrying various protective variants in *APOE, PCSK9*, and *APOC3*. Statistical significance was assessed using an ANOVA test.

Additionally, the presence of a loss-of-function mutation in the *PCSK9* gene or the null mutation R19* in *APOC3* was related to a low risk of atherosclerosis [16, 17]. First, our analysis did not discover the effect of the protective variants on telomere length when we compared the telomere length of the samples with various protective variants against their age and gender match controls (Supplementary Table 3 and Figure 4A). Additionally, individuals having different alleles of APOE had insignificant variation in telomere length (Figure 4B). However, comparing progeroid syndromes to samples with protective variants showed a significant difference in telomere length (*p* < 0.001). Individuals with protective variants have significantly longer telomere length than patients with progeroid syndrome and slightly longer telomeres when compared to controls (Figure 5).

## DISCUSSION

Progeroid syndromes are a set of genetic disorders characterized by heterogeneous symptoms, some of which mimic features of the normal aging process [9]. Therefore, many progeroid syndromes exhibit several hallmarks of aging, such as telomere attrition [13, 14, 18]. However, no specific mechanism exists to determine which aging hallmark is associated with each progeroid syndrome. Thus, we aimed to uncover whether telomere attrition is a common hallmark for all progeroid syndromes. Our results revealed significant telomere attrition in classical WS, CGL2, and DKC. On the other hand, we found no evidence for telomere attrition in HGPS, CRMCC, WRS, and atypical WS. Furthermore, we aimed to study the effect of specific protective variants on telomere length. Our findings revealed significant differences in telomere length between individuals with progeroid syndromes and those carrying protective variants.

WS is a segmental adult-onset progeroid syndrome with features of age acceleration such as hair graying and loss and skin atrophy. During mid-age, common age-related diseases might develop in WS patients, such as atherosclerosis and type 2 diabetes. WS is divided into classical and atypical based on the disease-causing mutation gene [19, 20]. An autosomal recessive variant in the *WRN* gene causes classical WS. However, atypical WS is more heterogeneous since the disease-causing variant might be located within any of the *LMNA, POLD1, SPRTN*, or *SAMHD1* genes [19, 20]. Our analysis showed that classical WS exhibits significant telomere attrition compared to atypical WS. In classical WS, the variant impacts the WRN protein and its role in telomere maintenance by destroying the WRN protein's ability to engage with the shelterin complex, a group of proteins that protect telomeric DNA. The loss of WRN's function in telomere maintenance among classical WS patients supports our finding of significant telomere attrition in classical WS patients with mutated WRN protein. Furthermore, it has been previously reported that the accelerated aging phenotype in WS patients is due to telomere dysfunction [21].

Atypical WS is a heterogenous disease diagnosed as WS in patients who do not carry *WRN* gene mutations [19]. Previous studies showed that mutated LMNA protein can enhance TRF2 protein degradation, where TRF2 is a subunit of the shelterin complex that protects telomeric DNA [22]. However, variants in other genes, such as *POLD1* and *SPRTN*, have no direct effect on telomere length. The *POLD1* protein is a subunit of DNA polymerase δ, which connects with the WRN helicase complex during DNA replication and repair; however, it has no role in telomere maintenance [20]. The SPRTN protein also has no role in telomere maintenance since it is part of the DNA repair process after replication [19]. Hence, our results showed no significant difference in telomere attrition between controls and patients with atypical WS.

Telomeropathies or telomere disorders are a group of rare genetic disorders with telomere attrition as their primary feature. Telomeropathies include dyskeratosis congenita (DKC), in which a disease-causing variant is often found within the dyskerin (*DKC1*) gene on the X chromosome or other telomere maintenance genes such as *TINF2*, *TERC*, *TERT*, *CTC1*, and other genes. A genetic variant in any of these genes results in reduced telomerase activity and shorter telomere length [23, 24]. To diagnose DKC, telomere length measurement is usually performed to differentiate between patients and normal individuals. In 2007, Alter et al. measured telomere length in blood leukocytes using flow fluorescence *in situ* hybridization (FISH) to confirm the diagnosis of 26 DKC patients, where they observed significant telomere attrition in these patients [25]. Even the change in the measurement technique for telomere length, such as using quantitative PCR to measure relative telomere length, showed considerable shortening in DKC patients compared to normal individuals [18]. These findings align with our results for DKC patients’ telomere shortening compared to the control. Since DKC is a heterogeneous disease with variants in different genes, earlier studies indicated that patients with *TINF2* variants have significantly shorter telomere lengths than patients with *DKC1* variants. Furthermore, they noted that telomere attrition depends on the gene, variant location, and disease severity, as they presented a single DKC patient with less severe symptoms with a specific variant in the *TINF2* gene with normal telomere length [26]. Further studies also demonstrated the dependence of telomere length on disease severity and variant type and effect while measuring telomere length in peripheral blood leukocytes from 65 cases of DKC using flow-FISH [27].

Berardinelli–Seip congenital lipodystrophy type 2 (CGL2) is an age acceleration disorder caused by variants in the *BSCL2* gene [28]. CGL2 is one of the lipodystrophies, a group of rare disorders with the typical characteristics of unrecovered adipose tissue loss. Lipodystrophies are widely known to include specific progeroid syndromes called lipodystrophy-associated progeroid syndromes [29]. To the best of our knowledge, our study is the first to report telomere attrition in CGL2 patients compared to controls. Further studies are needed to understand how BSCL2 variants are altering telomere length.

Several progeroid syndromes, such as HGPS, CRMCC, WRS, and atypical WS, didn’t show any evidence of telomere attrition during this study. Hutchinson-Gilford progeria syndrome (HGPS) is a rare premature aging disorder that influences children between 6 and 12 months, where they start to show symptoms of accelerated aging. Death of HGPS patients usually occurs between six and twenty years of age [30]. Classical HGPS is caused by disease-causing variants in the *LMNA* gene which encodes Lamin A protein, critical in the nuclear envelope architecture. Classical HGPS patients have a point mutation c.1824C>T that change mRNA splicing of the Lamin A gene, leading to progerin production [30]. In contrast, non-classical HGPS patients have the characteristic clinical features of HGPS but harbor the non-classic *LMNA* pathogenic variants [31]. Previous studies showed telomere attrition in progerin production-induced fibroblast cells when telomere length was measured using quantitative telomere peptide nucleic acid-FISH [32] or quantitative PCR [33]. Furthermore, using HGPS patients’ fibroblast, Decker et al. observed telomere shortening using quantitative-FISH. At the same time, they showed that hematopoietic cells, such as T cells, B cells, and NK cells obtained from HGPS patient samples, had normal telomere length compared to age-match controls [14]. Our analysis using blood samples from HGPS patients revealed no telomere attrition, consistent with the findings from Decker et al. This finding might be explained by the low or absent expression of the *LMNA* gene in hematopoietic cells, as shown by Decker et al. [14].

Cerebroretinal Microangiopathy with Calcifications and Cysts (CRMCC) is one of the rare telomeropathies caused by a variant in one of the telomere maintenance genes called conserved telomere maintenance component 1 (CTC1) [34, 35]. Hence, telomere attrition and defects in CRMCC patients are expected to cause the patient's clinical manifestations [9, 35]. Our analysis did not detect telomere shortening in CRMCC patients, which might be due to the small sample size. At the same time, the neonatal progeroid patients diagnosed with Wiedemann-Rautenstrauch Syndrome (WRS) did not show significant telomere shortening. Wiedemann-Rautenstrauch Syndrome is a heterogeneous disorder caused by a pathogenic variant found in one of a few genes, including *POLR3A*, *FBN1*, *CAV1*, and *SLC25A24* [36, 37]. Previous analyses by Korniszewski et al. were consistent with our findings, where they showed normal telomere length in WRS patient fibroblast when measured by terminal restriction fragment [38]. However, further studies are needed to investigate telomere length in WRS patients.

In this study, we investigated the effect of protective variants on telomere length to determine their role in maintaining telomere integrity. We found a tendency for telomere length increase in the protective group compared to controls (p=0.19, Figure 5) and a significant increase in telomere length when compared to progeroid syndromes (p < 0.001, Figure 5). A recent study also showed a significant effect of these protective variants on age acceleration, as these protective variants significantly decrease age acceleration when comparing antigeroid vs. progeroid syndrome groups [39].

We acknowledge some limitations in our current research study. First, the heterogeneity of our DNA methylation data included in this study. We collected publicly available DNA methylation data from multiple sources. The limited sample size is also considered one of the limitations of this study, which decreases the statistical power of our analysis. However, the rare incidence of the studied syndromes severely limits the number of diagnosed patients worldwide and, consequently, the availability of biological samples for validation purposes; for example, DKC is a very rare syndrome that influences 1 case per 1,000,000 individuals [40]. HGPS is also rare, with an estimated incidence of only one case in every 4 to 8 million newborns [41], and CGL2 is similarly uncommon, occurring in roughly one out of every 10 million individuals [42]. Finally, one limitation lies in the epigenetic calculator's inherent constraints to estimate the shortest telomeres’ length, which triggers replicative senescence [43–45]. This limitation is of particular interest as the length of the shortest telomeres determines cell fate through cellular senescence [45]. However, our methodology also has notable strength, specifically the ability to estimate the telomere length of large publicly available DNA methylation datasets [43].

Our analysis revealed that telomere shortening cannot be considered a common hallmark across all progeroid syndromes due to the variability in the genetic and molecular mechanisms driving these syndromes. These results suggest that therapeutic strategies targeting telomere maintenance should be tailored to the specific etiology of each syndrome. Our analysis also highlights the potential of using the epigenetic calculator in telomere length measurement to uncover telomere attrition. We demonstrated that the epigenetic calculator is an accurate tool for measuring telomere length, comparable to qPCR and FISH. In addition, we show the potential of non-invasive measurement of telomere length using an epigenetic calculator from blood samples. Additionally, to the best of our knowledge, this is the first study to estimate telomere length in various progeroid syndromes such as CGL2 and WRS and to confirm telomere attrition in specific progeroid syndromes. Finally, we have demonstrated the potential role of the protective variants in telomere length maintenance. However, further studies are needed to reveal the underlying mechanisms by which these protective variants maintain telomere length.

## MATERIALS AND METHODS

### Datasets and patient samples

Publicly available genome-wide DNA methylation data were collected for progeroid syndrome patients and controls from the Gene Expression Omnibus (GEO). DNA methylation data derived from human whole blood samples and profiled using different Illumina Infinium array platforms, including the EPIC and 450K arrays, were incorporated in this study. Using the GEO website, we identified five datasets with the following accession numbers (GSE182991, GSE131752, GSE100825, GSE75310, and GSE214297). In total, data from 57 progeroid syndrome patients diagnosed with WS, HGPS, CGL2, and DKC, as well as 42 healthy controls, were included in our study. Additionally, DNA methylation data for progeroid syndrome patients diagnosed with WRS and CRMCC, along with age and gender-matched healthy controls for WRS were generated in our lab, as shown in Supplementary Table 1. Informed consent was obtained from these patients, and necessary approval was taken from the institutional review board (IRB) at the University of Washington (STUDY00000233) and the QBRI IRB (QBRI-IRB 2019-029).

Additional data from our laboratory from individuals with known protective variants against premature aging disorders such as Alzheimer's and cardiovascular diseases were also included in this study. These protective variants are located within the APOE ε2, PCSK9 [*PCSK9* p.Cys679*], and *APOC3* c.55C>T (p.Arg19*) genes. As controls, 41 age and gender-matched samples who are carriers of the APOE ε3 allele were included. APOE ε3 allele is considered a neutral allele with no effect on the risk of developing Alzheimer's disease (AD).

### Profiling of DNA methylation

The Illumina Infinium MethylationEPIC BeadChip was used per the manufacturer's instructions to measure DNA methylation. Using EPIC v2.0, DNA methylation profiling for ~ 930k CpG sites was conducted on WRS samples and six matching controls provided by our laboratory. Furthermore, DNA methylation profiling using EPIC v1.0 was performed on two CRMCC patients. First, DNA quantity was assessed using Qubit™ 4 Fluorometer, and then 500 ng DNA was taken from each sample for bisulfite conversion using the EZ DNA Methylation Kit (Zymo Research, Irvine, CA, USA), followed by DNA amplification, fragmentation, and hybridization to the BeadChip. IDAT (intensity data) files were used for telomere length estimation. For Antigeroid samples, IDAT files were sourced from our previous publication performed on subjects with protective variants enrolled in the Qatar BioBank (https://www.researchsquare.com/article/rs-5304780/v1).

### Estimation of telomere attrition

DNA methylation data can be used to measure biological age and estimate telomere length [44, 46]. Thus, in this study, we used the DNA methylation age (DNAmAge) calculator from the Steve Horvath lab (https://dnamage.clockfoundation.org/) to estimate telomere length and biological ages, including Horvath Age, GrimAge, and PhenoAge for all the patients, controls, and protective samples. The DNAmAge calculator used normalized Beta values from GSE75310 and IDAT files from the rest of the data, normalized internally within the calculator using noob normalization to estimate telomere length and biological ages. Estimated telomere length, known as DNAmTLadjAge, is computed as the residual of regressing DNA methylation telomere length (DNAmTL) on actual age. In addition to age adjustment, our formula included gender adjustment as follows: lm(DNAmTL ~ Age + Gender, in contrast). Telomere attrition or telomere shortening is then indicated by the negative value of DNAmTLadjAge, while a positive value suggests a longer telomere length.

### Validation of telomere length

Quantitative RT-PCR was used to validate changes in the telomere length of DNA samples of Classical WS and CGL2 patients compared to matching (age, gender) healthy controls (Supplementary Table 2), using ScienCell's Absolute Human Telomere Length Quantification qPCR Assay Kit (#8918). The telomere primer set recognizes and amplifies telomere length by normalizing the target samples to reference genomic DNA provided in the kit. It has a 100bp telomere sequence located on human chromosome 17. Two qPCR reactions were performed for each DNA sample: one with single-copy reference (SCR) primer and the other one with the telomere primer. Both PCRs were performed as follows: 1 μL of reference or genomic DNA samples from patients and controls (5-10 ng/μL), 2 μL telomere primer, 10 μL of 2x qPCR master mix, and 7 μL nuclease-free water in a final volume of 20 μL. The qPCR reactions were optimized for the individual target genes using the Quant Studio 6 Flex System. All reactions were performed in triplicate. Amplification was performed following the manufacturer's instructions: denaturation for 10 min at 95° C followed by 32 cycles of denaturation for 20 s at 95° C, annealing for 20 s at 52° C, and extension for 45 s at 72° C. The reference genomic DNA sample with known telomere length was used as a reference for calculating the absolute telomere length of target samples [47, 48]. Finally, absolute telomere length was used to investigate telomere attrition by computing the residual of regressing DNA absolute telomere length on actual age while adjusting for gender.

### Statistical analysis

All statistical tests were conducted on RStudio version 4.1.1. To compare age and gender differences between the control and patient groups, we conducted a nonparametric Wilcoxon test for age and chi-square tests for gender. For multiple group comparisons between the different protective variants and their control, the parametric test ANOVA and the Fisher test with Monte Carlo simulation were used for age and gender, respectively. To calculate the significant difference in telomere length between controls, progeroid, and protective groups, we combined all controls and excluded progeroid patients younger than 10 years old to avoid significant age differences. Furthermore, we used the ggpubr package to visualize the data and calculate all the significant differences in the various comparisons. Several statistical tests were conducted to evaluate telomere length differences based on the data's distribution and the number of comparisons. These tests included the Kruskal-Wallis test, ANOVA, T-test, and Wilcoxon test. All comparisons with *p*-values less than 0.05 were considered statistically significant.

## Supplementary Material

Supplementary Figure

Supplementary Tables
