# Supplementary material for: Investigating telomere length in progeroid syndromes: implications for aging disorders
Source: Aging (Albany NY). 2025 May 28;17(5):1190–205. doi: 10.18632/aging.206255 (PMC12151510; doi:10.18632/aging.206255)
Supplement: Supplementary Figure [file aging-17-206255-s001.pdf]

SUPPLEMENTARY FIGURE

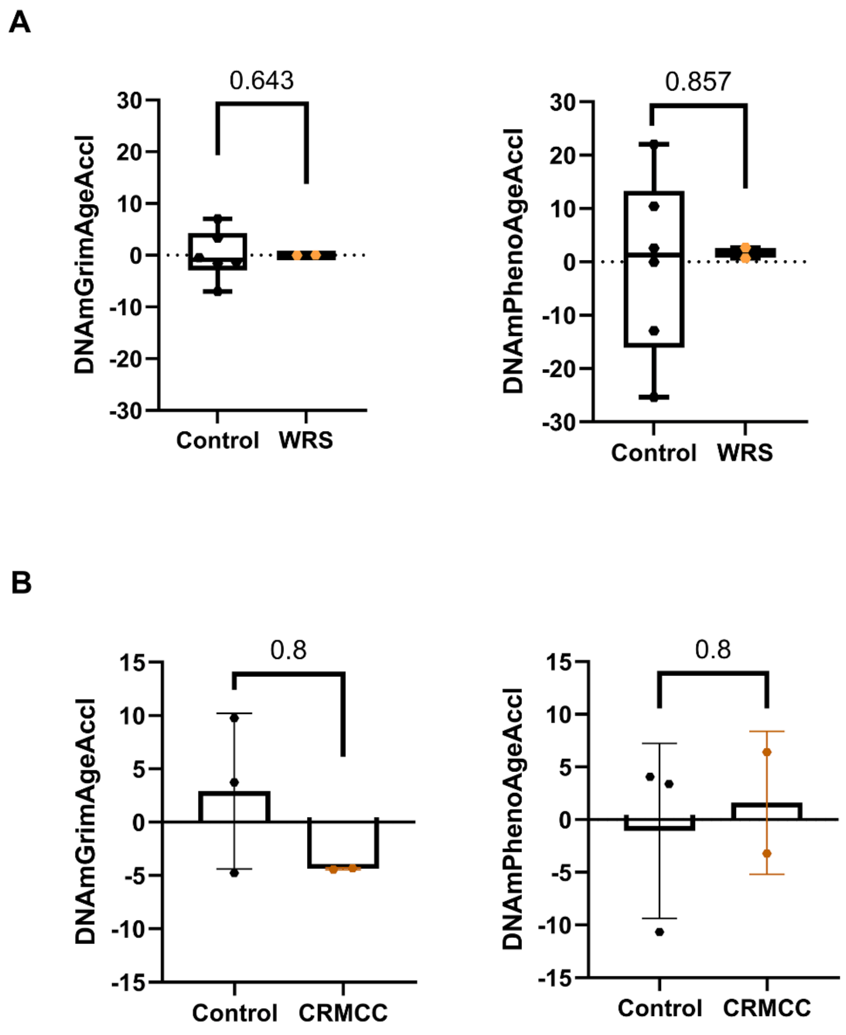

Supplementary Figure 1. Epigenetic age acceleration between controls and WRS patients. (**A**) Boxplots display epigenetic age acceleration, measured using the GrimAge and PhenoAge clocks, showing no significant differences between controls (n = 6) and WRS patients (n =2). Statistical significance was assessed using a two-tailed Wilcoxon rank-sum test and Student's t-test for GrimAge and PhenoAge clocks, respectively. Epigenetic age acceleration between controls (n = 3) and CRMCC patients (n = 2): (**B**) Boxplots with individual data points illustrate insignificant differences in epigenetic age acceleration, as measured using the GrimAge and PhenoAge clocks. Statistical significance was assessed using a two-tailed Wilcoxon rank-sum test.
