# Supplementary material for: Investigating telomere length in progeroid syndromes: implications for aging disorders
Source: Aging (Albany NY). 2025 May 28;17(5):1190–205. doi: 10.18632/aging.206255 (PMC12151510; doi:10.18632/aging.206255)
Supplement: Supplementary Tables [file aging-17-206255-s002.pdf]

SUPPLEMENTARY TABLES

Supplementary Table 1. Progeroid syndrome patients and controls included in telomere length estimation.

GEO Accession	Sample ID	Condition	Disease	Age (year)	Gender	Platform	Reference
GSE100825	GSM2694066	Progeroid	Classical WS	51	male	Infinium MethylationEPIC	[1]
	GSM2694067	Control	NA	44	male	Infinium MethylationEPIC	
	GSM2694068	Progeroid	Classical WS	53	female	Infinium MethylationEPIC	
	GSM2694069	Control	NA	53	female	Infinium MethylationEPIC	
	GSM2694070	Progeroid	Classical WS	44	male	Infinium MethylationEPIC	
	GSM2694071	Control	NA	52	male	Infinium MethylationEPIC	
GSE182991	GSM5548192	Progeroid	Classical HGPS	9.2	female	Infinium MethylationEPIC	[2, 3]
	GSM5548193	Progeroid	Classical HGPS	2.2	male	Infinium MethylationEPIC	
	GSM5548194	Progeroid	Classical HGPS	0.8	male	Infinium MethylationEPIC	
	GSM5548195	Progeroid	Classical HGPS	1.5	female	Infinium MethylationEPIC	
	GSM5548196	Progeroid	Classical HGPS	3.7	male	Infinium MethylationEPIC	
	GSM5548197	Progeroid	Classical HGPS	7.8	female	Infinium MethylationEPIC	
	GSM5548198	Progeroid	Classical HGPS	0.3	female	Infinium MethylationEPIC	
	GSM5548199	Progeroid	Classical HGPS	1.4	male	Infinium MethylationEPIC	
	GSM5548200	Progeroid	Non-Classical HGPS	11.3	female	Infinium MethylationEPIC	
	GSM5548201	Progeroid	Non-Classical HGPS	17.3	male	Infinium MethylationEPIC	
	GSM5548202	Progeroid	Non-Classical HGPS	6.2	male	Infinium MethylationEPIC	
	GSM5548203	Progeroid	Non-Classical HGPS	5.3	male	Infinium MethylationEPIC	
	GSM5548204	Progeroid	Non-Classical HGPS	0.7	male	Infinium MethylationEPIC	
	GSM5548205	Progeroid	Non-Classical HGPS	41	female	Infinium MethylationEPIC	
	GSM5548206	Progeroid	Non-Classical HGPS	4.6	male	Infinium MethylationEPIC	
	GSM5548207	Control	NA	8.2	female	Infinium MethylationEPIC	
	GSM5548208	Control	NA	1	male	Infinium MethylationEPIC	
	GSM5548209	Control	NA	0.4	male	Infinium MethylationEPIC	
	GSM5548210	Control	NA	2.8	female	Infinium MethylationEPIC	
	GSM5548211	Control	NA	4	male	Infinium MethylationEPIC	
	GSM5548212	Control	NA	8.1	female	Infinium MethylationEPIC	
	GSM5548213	Control	NA	2.7	female	Infinium MethylationEPIC	
	GSM5548214	Control	NA	0.7	male	Infinium MethylationEPIC	
	GSM5548215	Control	NA	15.9	male	Infinium MethylationEPIC	
	GSM5548216	Control	NA	4	male	Infinium MethylationEPIC	
	GSM5548217	Control	NA	39.4	female	Infinium MethylationEPIC	
	GSM5548218	Control	NA	4.7	female	Infinium MethylationEPIC	
GSE214297	GSM6603331	Progeroid	CGL2	1	female	Infinium MethylationEPIC	[3]
	GSM6603332	Progeroid	CGL2	2	female	Infinium MethylationEPIC	
	GSM6603333	Progeroid	CGL2	4	female	Infinium MethylationEPIC	
	GSM6603334	Progeroid	CGL2	5	male	Infinium MethylationEPIC	
	GSM6603335	Progeroid	CGL2	7	female	Infinium MethylationEPIC	
	GSM6603336	Progeroid	CGL2	3	female	Infinium MethylationEPIC	
	GSM6603337	Progeroid	CGL2	19	male	Infinium MethylationEPIC	
	GSM6603342	Control	NA	8	female	Infinium MethylationEPIC	
	GSM6603345	Control	NA	6	female	Infinium MethylationEPIC	
	GSM6603346	Control	NA	23	male	Infinium MethylationEPIC	
	GSM3815066	Control	NA	30	female	Infinium MethylationEPIC	
GSE131752	GSM3815067	Progeroid	Atypical WS	30	female	Illumina Infinium HumanMethylation850 BeadChip	[4]
	GSM3815068	Control	NA	37	male	Illumina Infinium HumanMethylation850 BeadChip	
	GSM3815069	Progeroid	Classical WS	37	male	Illumina Infinium HumanMethylation850 BeadChip	
	GSM3815070	Control	NA	9	female	Illumina Infinium HumanMethylation850 BeadChip	
	GSM3815071	Progeroid	Atypical WS	9	female	Illumina Infinium HumanMethylation850 BeadChip	
	GSM3815072	Control	NA	45	male	Illumina Infinium HumanMethylation850 BeadChip	
	GSM3815073	Progeroid	Classical WS	45	male	Illumina Infinium HumanMethylation850 BeadChip	

	GSM3815074	Progeroid	Classical WS	39	male	Illumina Infinium HumanMethylation850 BeadChip	
	GSM3815075	Control	NA	39	male	Illumina Infinium HumanMethylation850 BeadChip	
	GSM3815076	Progeroid	Atypical WS	37	female	Illumina Infinium HumanMethylation850 BeadChip	
	GSM3815077	Control	NA	37	female	Illumina Infinium HumanMethylation850 BeadChip	
	GSM3815078	Progeroid	Classical WS	47	male	Illumina Infinium HumanMethylation850 BeadChip	
	GSM3815079	Control	NA	47	male	Illumina Infinium HumanMethylation850 BeadChip	
	GSM3815080	Progeroid	Atypical WS	13	female	Illumina Infinium HumanMethylation850 BeadChip	
	GSM3815081	Control	NA	13	female	Illumina Infinium HumanMethylation850 BeadChip	
	GSM3815082	Control	NA	40	male	Illumina Infinium HumanMethylation850 BeadChip	
	GSM3815083	Progeroid	Classical WS	40	male	Illumina Infinium HumanMethylation850 BeadChip	
	GSM3815084	Control	NA	30	female	Illumina Infinium HumanMethylation850 BeadChip	
	GSM3815085	Progeroid	Atypical WS	30	female	Illumina Infinium HumanMethylation850 BeadChip	
	GSM3815086	Control	NA	36	male	Illumina Infinium HumanMethylation850 BeadChip	
	GSM3815087	Progeroid	Atypical WS	36	male	Illumina Infinium HumanMethylation850 BeadChip	
	GSM3815088	Control	NA	49	male	Illumina Infinium HumanMethylation850 BeadChip	
	GSM3815089	Progeroid	Classical WS	49	male	Illumina Infinium HumanMethylation850 BeadChip	
	GSM3815090	Progeroid	Classical WS	18	male	Illumina Infinium HumanMethylation850 BeadChip	
	GSM3815091	Control	NA	18	male	Illumina Infinium HumanMethylation850 BeadChip	
	GSM3815092	Progeroid	Classical WS	43	male	Illumina Infinium HumanMethylation850 BeadChip	
	GSM3815093	Control	NA	43	male	Illumina Infinium HumanMethylation850 BeadChip	
	GSM3815094	Progeroid	Classical WS	37	male	Illumina Infinium HumanMethylation850 BeadChip	
	GSM3815095	Control	NA	37	male	Illumina Infinium HumanMethylation850 BeadChip	
	GSM3815096	Progeroid	Classical WS	31	female	Illumina Infinium HumanMethylation850 BeadChip	
	GSM3815097	Control	NA	31	female	Illumina Infinium HumanMethylation850 BeadChip	
	GSM3815098	Control	NA	37	female	Illumina Infinium HumanMethylation850 BeadChip	
	GSM3815099	Progeroid	Classical WS	37	male	Illumina Infinium HumanMethylation850 BeadChip	
	GSM3815100	Control	NA	43	male	Illumina Infinium HumanMethylation850 BeadChip	
	GSM3815101	Progeroid	Classical WS	43	male	Illumina Infinium HumanMethylation850 BeadChip	
	GSM3815102	Control	NA	22	male	Illumina Infinium HumanMethylation850 BeadChip	
	GSM3815103	Progeroid	Classical WS	22	male	Illumina Infinium HumanMethylation850 BeadChip	
	GSM3815104	Control	NA	59	male	Illumina Infinium HumanMethylation850 BeadChip	
	GSM3815105	Progeroid	Classical WS	59	female	Illumina Infinium HumanMethylation850 BeadChip	
	GSM3815106	Progeroid	Classical WS	45	male	Illumina Infinium HumanMethylation850 BeadChip	
	GSM3815107	Control	NA	45	male	Illumina Infinium HumanMethylation850 BeadChip	
	GSM3815108	Progeroid	Classical WS	32	male	Illumina Infinium HumanMethylation850 BeadChip	
	GSM3815109	Control	NA	32	male	Illumina Infinium HumanMethylation850 BeadChip	
	GSM3815110	Progeroid	Classical WS	36	female	Illumina Infinium HumanMethylation850 BeadChip	
	GSM3815111	Control	NA	36	female	Illumina Infinium HumanMethylation850 BeadChip	
	GSM3815112	Progeroid	Classical WS	38	male	Illumina Infinium HumanMethylation850 BeadChip	
	GSM3815113	Control	NA	38	male	Illumina Infinium HumanMethylation850 BeadChip	
GSE75310	GSM1949187	Progeroid	DKC	16	female	Illumina HumanMethylation450 BeadChip	[5]
	GSM1949188	Progeroid	DKC	2	female	Illumina HumanMethylation450 BeadChip	
	GSM1949189	Progeroid	DKC	3	female	Illumina HumanMethylation450 BeadChip	
	GSM1949190	Progeroid	DKC	10	female	Illumina HumanMethylation450 BeadChip	
In-house	BB1010	Progeroid	CRMCC	39	male	Infinium MethylationEPIC V2	
	BB1070	Progeroid	CRMCC	18	male	Infinium MethylationEPIC V2	
In-house	131575	Control	NA	4	female	Infinium MethylationEPIC V2	
	24.028	Progeroid	WRS	0.3	female	Infinium MethylationEPIC V2	
	24.054	Progeroid	WRS	0.3	female	Infinium MethylationEPIC V2	
	KNN	Control	NA	6	male	Infinium MethylationEPIC V2	

Supplementary References

1. Guastafierro T, Bacalini MG, Marcoccia A, Gentilini D, Pisoni S, Di Blasio AM, Corsi A, Franceschi C, Raimondo D, Spanò A, Garagnani P, Bondanini F. Genome-wide DNA methylation analysis in blood cells from patients with Werner syndrome. Clin Epigenetics. 2017; 9:92. https://doi.org/10.1186/s13148-017-0389-4 PMID:28861129

2. Bejaoui Y, Razzaq A, Yousri NA, Oshima J, Megarbane A, Qannan A, Potabattula R, Alam T, Martin GM, Horn HF, Haaf T, Horvath S, El Hajj N. DNA methylation signatures in Blood DNA of Hutchinson-Gilford Progeria syndrome. Aging Cell. 2022; 21:e13555. https://doi.org/10.1111/acel.13555 PMID:35045206

3. Qannan A, Bejaoui Y, Izadi M, Yousri NA, Razzaq A, Christiansen C, Martin GM, Bell JT, Horvath S, Oshima J, Megarbane A, Ericsson J, Pourkarimi E, El Hajj N. Accelerated epigenetic aging and DNA methylation alterations in Berardinelli-Seip congenital lipodystrophy. Hum Mol Genet. 2023; 32:1826–35. https://doi.org/10.1093/hmg/ddad016 PMID:36715159

4. Maierhofer A, Flunkert J, Oshima J, Martin GM, Poot M, Nanda I, Dittrich M, Müller T, Haaf T. Epigenetic signatures of Werner syndrome occur early in life and are distinct from normal epigenetic aging processes. Aging Cell. 2019; 18:e12995. https://doi.org/10.1111/acel.12995 PMID:31259468

5. Weidner CI, Lin Q, Birkhofer C, Gerstenmaier U, Kaifie A, Kirschner M, Bruns H, Balabanov S, Trummer A, Stockklausner C, Höchsmann B, Schrezenmeier H, Wlodarski M, et al. DNA methylation in PRDM8 is indicative for dyskeratosis congenita. Oncotarget. 2016; 7:10765–72. https://doi.org/10.18632/oncotarget.7458 PMID:26909595

Supplementary Table 2. Samples included in the validation using Quantitative PCR.

Sample ID	Condition	Age (year)	Gender
LGA	CGL2	3	Female
ESA	CGL2	19	Male
CEKA	CGL2	20	Male
PWM-18	Classical WS	18	Male
PWM-32	Classical WS	32	Male
PWM-36	Classical WS	36	Female
PWM-43-1	Classical WS	43	Male
PWM-45-1	Classical WS	45	Male
PWM-47	Classical WS	47	Male
PWM-49	Classical WS	49	Male
YGN	Control	6	Female
CSN	Control	23	Male
REKN	Control	21	Female
CM-18	Control	18	Male
CM-32	Control	32	Male
CM-36	Control	36	Female
CM-43-1	Control	43	Male
CM-45-1	Control	45	Male
CM-47	Control	47	Male
CM-49	Control	49	Male

Supplementary Table 3. Demographics of protective and control samples.

	**Control**	***APOE***	***APOC3***	***PCSK9***	**P-value**
N	41	21	6	9	
Age, median (IQR)	37 (29-45)	37 (31-44)	37.5 (31.25-40)	50 (30-57)	0.49
Gender					0.89
Male	8	6	1	2	
Female	33	15	5	7	

Note: p-values are significant at less than 0.05.
For Age: p-value was calculated using the parametric test ANOVA.
For Gender: p-value was calculated using the Fisher test with a Monte Carlo simulation to estimate the p-value for more than two groups.
